# Supplementary material for: Outcomes in Patients with Pulmonary Arterial Hypertension Underwent Transcatheter Closure of an Atrial Septal Defect
Source: J Clin Med. 2023 Mar 28;12(7):2540. doi: 10.3390/jcm12072540 (PMC10095540; doi:10.3390/jcm12072540)

Supplementary Table S1. Summary of Patients on PAH treatment after device closure of atrial septal defects with severe PAH.

Patient No	Age	Gender	Treatment strategy	Final PAH	Medication to closure (months)	F/U duration after closure (months)	AFib	PAH medication
1	34.9	F	Treat and repair	Severe	37	92	No	Macitentan, Sildenafil, Selexipag
2	66.0	F	Treat and repair	Severe	12	138	RFCA → No	Macitentan, Sildenafil, Selexipag
3	32.1	F	Treat and repair	Severe	10	96	No	Macitentan, Sildenafil
4	26.0	F	Treat and repair	Severe	8	150	No	Macitentan, Sildenail
5	60.0	F	Treat and repair	Moderate	12	75	RFCA → No	Sildenafil, Selexipag
6	20.0	F	Closure and medication	Moderate	NA	60	No	Macitentan, Selexipag
7	36.5	F	Closure and medication	Mild	NA	95	No	Bosentan →Macitentan
8	60.0	F	Closure and medication	Mild	NA	84	No	Bosentan →Macitentan
9	30.0	F	Closure and medication	Mild	NA	60	No	Sildenafil →Macitentan
10	28.0	M	Closure and medication	Normalized	NA	76	No	Macitentan → No
11	51.0	M	Closure and medication	Normalized	NA	84	Yes, on AAD	Macitentan →No
12	65.0	M	Closure and medication	Normalized	NA	64	Yes, on AAD	Macitentan →No
13	52.8	M	Closure and medication	Normalized	NA	72	Yes, on AAD	Macitentan→ No
14	30.3	F	Closure and medication	*Death*	NA	65	No	Sidenafil, Bosentan, Iloprost

AAD, antiarrhythmic drug; AFib, atrial fibrillation; F, female; F/U, follow-up; NA, not applicable; PAH, pulmonary arterial hypertension; RFCA, radio frequency catheter ablation

Supplementary Figure S1. Treatment in patients with atrial fibrillation

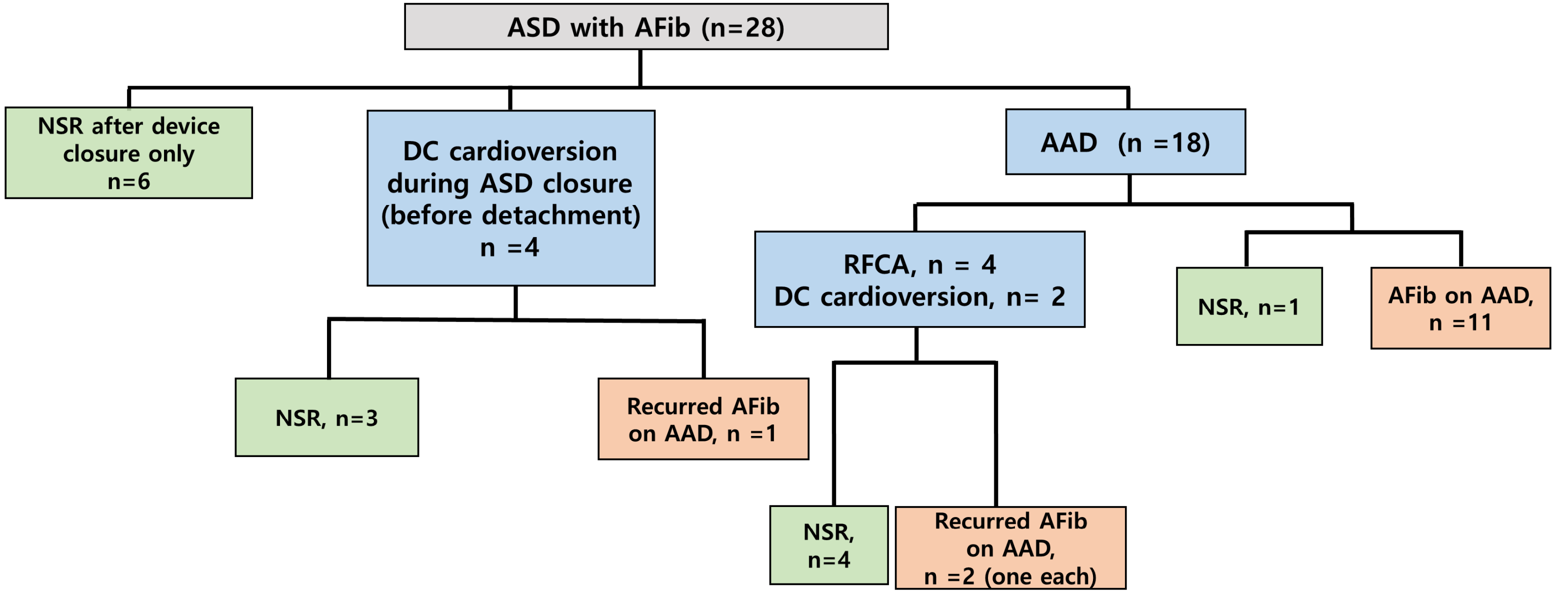

AAD, antiarrhythmic drug; AFib, atrial fibrillation; ASD, atrial septal defect; NSR, normal sinus rhythm

Supplementary Figure S2. Clinical outcome after transcatheter closure for atrial septal defects

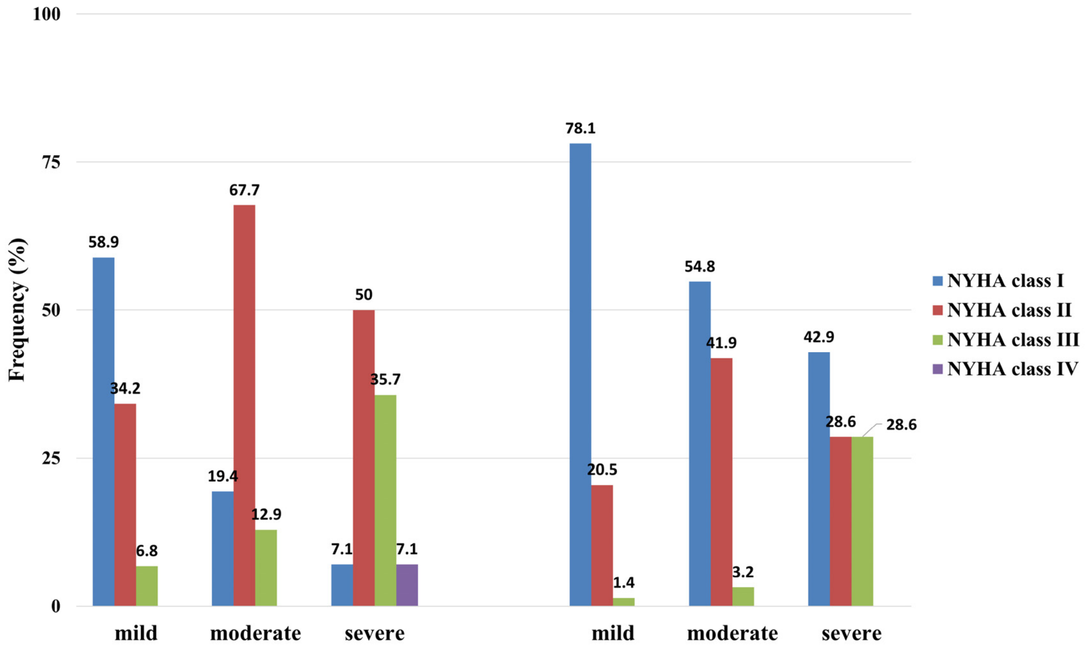

NYHA, New York Heart Association Classification

Supplementary Figure S3. Treatment outcomes of severe pulmonary arterial hypertension patients

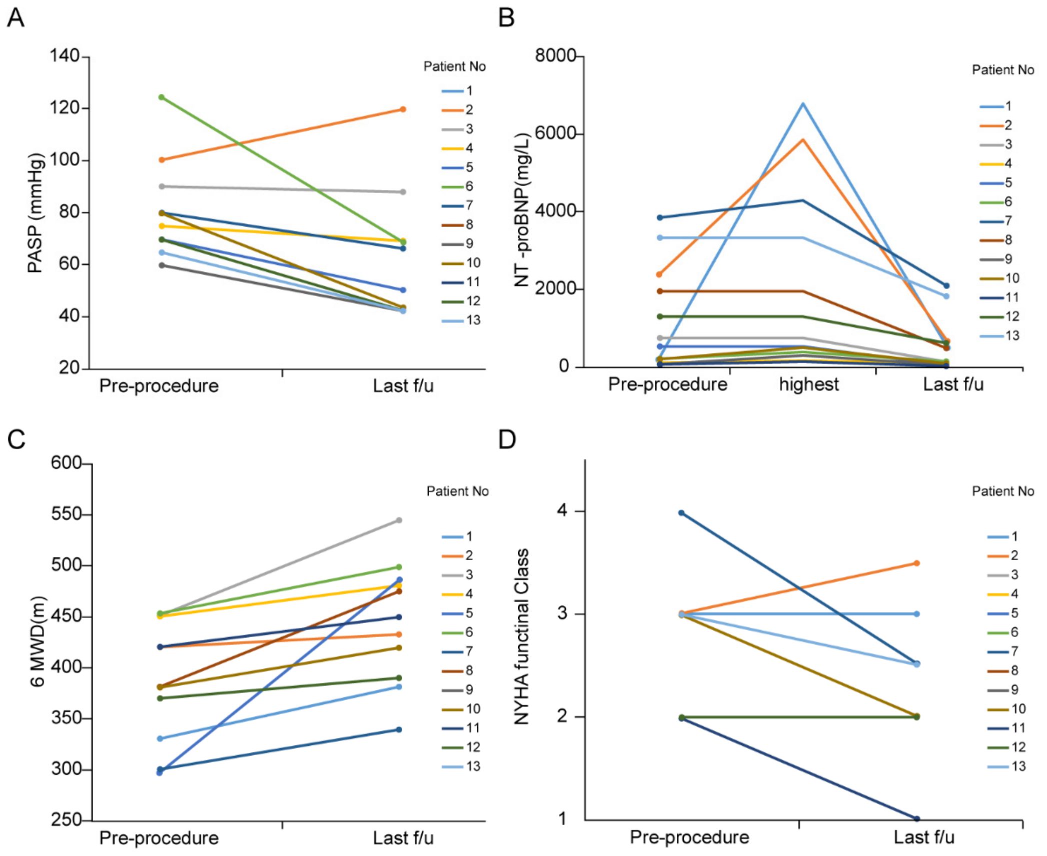

f/u, follow-up; No, number; NT-proBNP, N-terminal pro B-type natriuretic peptide; PASP, pulmonary arterial systolic pressure, NYHA, New York Heart Association; 6MWD, 6-minute walking distance

Supplementary Figure S4. ROC curve analysis to predict rPAH post-tcASD closure

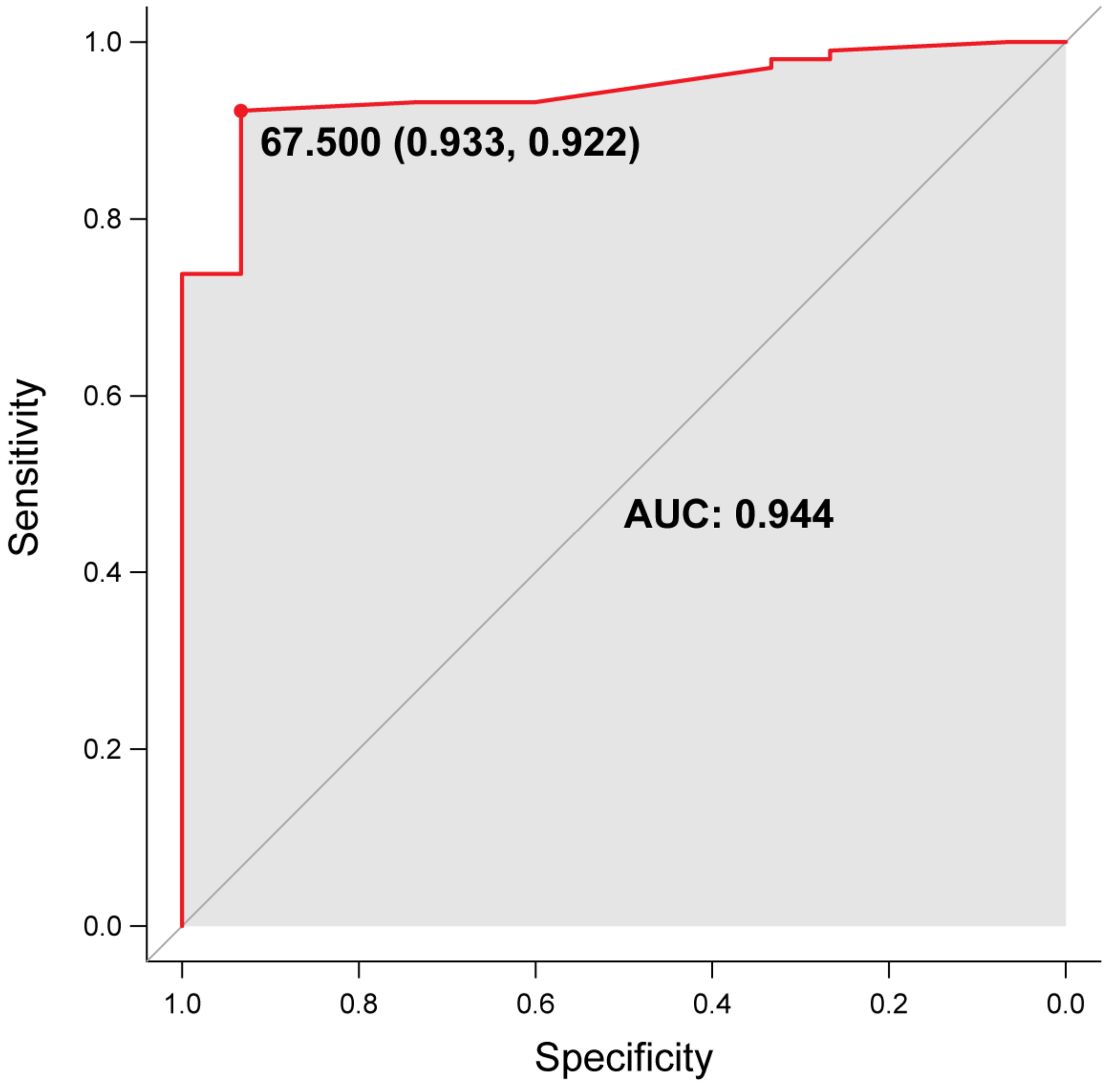

Supplement: Supplementary file 1 [file jcm-12-02540-s001.zip › jcm-2244878-supplementary.pdf]
